# Differences in rhizosphere soil fungal communities of wild and cultivated *Paeonia ludlowii* species

**DOI:** 10.3389/fpls.2023.1194598

**Published:** 2023-09-11

**Authors:** Hongyong Qiao, Danlei Gao, Tao Yuan

**Affiliations:** ^1^ Beijing Key Laboratory of Ornamental Plants Germplasm Innovation & Molecular Breeding, National Engineering Research Center for Floriculture, Beijing Laboratory of Urban and Rural Ecological Environment, Key Laboratory of Genetics and Breeding in Forest Trees and Ornamental Plants of Ministry of Education, School of Landscape Architecture, Beijing Forestry University, Beijing, China; ^2^ Science and Technology Development Center, National Forestry and Grassland Administration, Beijing, China

**Keywords:** *Paeonia ludlowii*, wild species, cultivated species, rhizosphere soil fungi, molecular network

## Abstract

**Introduction:**

*Paeonia ludlowii* is a rare and endangered plant species with a high application value. However, its low cultivation success rate in China has severely limited its protection, development, and utilization. In addition to natural factors, microorganisms in the rhizosphere play an important role in determining its cultivation success.

**Methods:**

In this study, growth indexes and soil physicochemical properties of both wild (origin: Nyingchi) and cultivated (introduction: Luanchuan) species of *P. ludlowii* were measured during the flowering, fruiting, and autumn foliage stages. ITS high-throughput sequencing technology was employed to detect rhizosphere soil fungi, and the diversity, community structure, functional prediction, molecular network, and ecological processes of the microbial community assembly were examined by multidirectional analysis.

**Results and discussion:**

The results indicated that: both wild and cultivated *P. ludlowii* species were able to flower and fruit normally, although the wild species had a higher number of flowers and fruits and higher soil available phosphorus and available potassium contents than those of the cultivated species. Ascomycota and Basidiomycota were the dominant rhizosphere soil fungal phyla in both *P. ludlowii* species. However, our network analysis showed that Ascomycota as the key fungal phylum of the wild species, whereas the cultivated species lacked key fungi. The community assembly mechanisms of rhizosphere soil fungi in both wild and cultivated species were primarily stochasticity, with no significant differences between them. Based on the results of FUNGuild and molecular network analyses, cultivated species had a higher proportion of fungi, such as Soil Saprotroph, that can easily cause diseases. Additionally, the network connections among fungi were weaker in the cultivated species than those in the wild species, which increased the cultivated species susceptibility to external environmental interferences. Therefore, from a soil microorganism perspective, this study suggests that, after the introduction and cultivation of *P. ludlowii*, if rhizosphere soil fungi fail to gradually form a close network relationship and instead promote the growth of pathogenic fungi, the fungal ecosystem would become vulnerable.

## 1 Introduction

*Paeonia ludlowii* is a tall deciduous shrub belonging to the Paeoniaceae family and *Paeonia* genus. Flowers typically appear at the top of (two) three or four branches or on the leaf axils. The petals, filaments and anthems are pure yellow and can be stably inherited, making them highly valuable for breeding and ornamental purposes (Editorial Committee of Flora, Chinese Academy of Sciences, 2006). Additionally, the flowers, roots, and seeds of *P. ludlowii* can be developed and utilized (Li and Wang, 2019; Zhang et al., 2020; Lu et al., 2021). Owing to its narrow distribution, poor natural regeneration ability, sparse population, and habitat degradation and loss, *P. ludlowii* has been included in China’s “List of National Secondary Protected Plants” (National Forestry and Grassland Administration, 2021).

Despite self-pollination and outcrossing, the seed setting rate for *P. ludlowii* in its country of origin is a mere 29.01% (Tang et al., 2021); furthermore, seedlings take 2–3 years to emerge, with a low emergence rate of only 2.3–4.0% (Yang et al., 2007; Hao et al., 2014; Qiu et al., 2016); furthermore, the seedling stage is prolonged, taking 4–6 years for the plant to bloom and bear fruit (Yang, 2010). Currently, the population of *P. ludlowii* is declining, and is largely sustained by elderly individuals (Yang et al., 2007). Recent studies have revealed that the abortion of *P. ludlowii* occurs during the process of double fertilization and ovule development, and its low seed-setting rate is a key limiting factor for the expansion of wild populations, as well as introduction and cultivation efforts (Chen et al., 2022; Chen et al., 2023).Since the late 20th century, regions in China including Shandong, Henan, and Beijing have attempted to introduce and cultivate *P. ludlowii*, however, most areas experienced plant death after introduction, failed to bloom, or exhibited rare and unstable flowering stages. As of now, only a few regions, such as Luoyang Paeoniaceae Ex-situ Protection Center in Henan Province (Luanchuan), Gansu Provincial Forestry Science and Technology Extension Station (Lanzhou), and Vegetable Research Institute of Agriculture and Animal Husbandry College of Tibet Autonomous Region (Lhasa), have successfully introduced varieties that can bloom and bear fruit under normal circumstances (Ni, 2009; Li et al., 2011; Cui et al., 2019).

Plants and microorganisms co-evolve, and the stability of microorganisms is crucial to the adaptability of plants (Hassani et al., 2019). Rhizosphere soil fungi perform a range of vital ecological functions, and play key roles in improving plant nutrient absorption, organic matter decomposition, and pathogen control (Raaijmakers et al., 2009; Garcia et al., 2016). They can directly affect plant growth through mutualism or pathogenicity, or indirectly affect plant growth by affecting soil nutrient availability and cycling (Wagg et al., 2014; Hannula et al., 2017). At the same time, fungal communities are more sensitive to changes in habitat, and their diversity and community structure are often closely related to environmental factors such as soil physicochemical properties (non-biological factors) and plant communities (biological factors) (Rouphael et al., 2015; Frąc et al., 2018). Introduction and cultivation in different regions are important measures for protecting endangered plants and expanding their ecological adaptability. Factors such as light, temperature, precipitation, and soil quality affect the success of introduction and cultivation. In the past, researchers have mainly focused on changes in the aboveground yield and plant phenotypes after introduction and cultivation. However, in recent years, researchers have gradually realized that rhizosphere soil microorganisms also directly or indirectly affect introduction and cultivation in various ways (Pérez-Jaramillo et al., 2016).

Currently, there is only one study on the rhizosphere microbial diversity and community structure of *P. ludlowii* in the introduction area (Gao et al., 2022). The dominant groups in the rhizosphere soil were Proteobacteria (bacteria), Ascomycota and Basidiomycota (fungi), *Claroideoglomus* and *Septoglomus* (arbuscular mycorrhizal fungi [AMF]). Additionally, the contents of soil organic matter, ammonium nitrogen, available potassium (AK) and pH of the introduction region were significantly correlated with the rhizosphere soil microbial community. Previous studies have shown that the dominant rhizosphere soil fungi in the original Nyingchi region are Ascomycota and Basidiomycota, and the dominant rhizosphere soil bacteria are Proteobacteria and Acidobacteria (Lu et al., 2020a; Lu et al., 2020b). However, to date, there has been no relevant research on the comparison of rhizosphere soil fungi between wild and cultivated *P. ludlowii* species; therefore, it is not possible to discern the differences in rhizosphere soil fungi between the origin and introduction regions, nor is it possible to speculate on the reasons for the failure of *P. ludlowii* introduction from the perspective of soil fungi in other areas.

In response to the current difficulties in the introduction and cultivation of *P. ludlowii*, this study focused on rhizosphere soil fungi and combined the three key stages of growth and development during the lifespan of *P. ludlowii*: flowering, fruiting, and autumn foliage stages. We concurrently measured the growth indexes and physicochemical properties of the soil. This study aimed to (1) investigate the diversity and structural differences in rhizosphere soil fungal communities between wild and cultivated species of *P. ludlowii*, (2) evaluate the factors influencing rhizosphere soil fungi of wild and cultivated species of *P. ludlowii*, and (3) reveal the molecular network characteristics and ecological assemblage process of rhizosphere soil fungi of wild and cultivated *P. ludlowii* species. This study provides a new theoretical basis for improving the success rate of the introduction and cultivation of *P. ludlowii* in other areas by developing and utilizing its microbial resources, as well as providing a reference for the ex-situ protection of other endangered plants.

## 2 Material and methods

### 2.1 Site description and sample collection

*P. ludlowii* is naturally found in the region spanning from Linzhi City to Shannan City in the Tibetan Autonomous Region of China. The wild species studied in this research are situated in Qunigongga Village, Bayi District, Linzhi City (29°50’44”N, 94°63’56”E), at an altitude of 3,046 m with a temperate monsoon climate. The average annual precipitation is 702.1 mm, the average annual relative humidity is 71%, the average annual temperature is 8.7°C, and sunshine hours are 3,019.5 h. The soil is sandy loam. This wild population thrives on roadside forest margins, sunny slopes, and middle slopes. There were approximately 300 plants in this population, all of which grew well (**Figure S1**).

The cultivated species *P. ludlowii* can be obtained from Luoyang Paeoniaceae Ex-situ Protection Center, located in Xiaohong Village, Luanchuan County, Luoyang City, Henan Province (33°56’05”N, 111°21’36”E) (**Figure S2**) at an altitude of 1,240 m with a continental monsoon climate. The average annual precipitation is 800–1,000 mm, average annual relative humidity is 66%, average annual temperature is 14.5°C, and sunshine hours are 2,301.7 h, and the soil is sandy clay. Currently, more than 200 flowering plants of *P. ludlowii* were sown and bred in the autumn of 2002. Seeds were collected from Nyingchi City wild species of *P. ludlowii*, which first bloomed in 2008. Following harvesting seeds and raising seedlings, the flowering plants bloom and bear fruit normally (Wang et al., 2013). Since 2015, the introduction region has been poorly managed, with weed removal taking place only once a year in the summer and no fertilization.

Fixed sample plots were established based on the terrain and topography at predetermined sampling locations. This study focused on the following three stage: flowering, fruiting and autumn foliage, and the sampling time was determined according to the phenological observation results of Xue and Luo (2014). The collection time of wild species of *P. ludlowii* in Nyingchi City was 2021-5-8(LZT1), 7-6(LZT2) and 9-8(LZT3), and cultivated species of *P. ludlowii* in Luanchuan County was 2021-5-17(LCT1), 7-17(LCT2) and 9-13(LCT3). *P. ludlowii* has a robust fleshy root system with dense absorbent roots on its surface, which are densely distributed near the surface, therefore, soil sampling was conducted at a depth of 0–20 cm (Gao et al., 2022). In each habitat sample plot, five *P. ludlowii* plants with similar growth patterns, spaced > 3 m apart, were selected as the five biological replicates. Three sampling points were established at a distance of 30 cm from the base of each *P. ludlowii*. After removing surface litter, soil samples were collected using a soil drill. At the laboratory, roots were removed from each sample and large pieces of attached soil were shaken off. The rhizosphere soil attached to the root surfaces was slightly brushed off. Thirty samples were collected from the two regions and divided into two parts. One part was stored at -80°C for microbial sequencing, and the other part was dried to determine soil physicochemical properties.

### 2.2 DNA extraction, PCR amplification, and Illumina MiSeq

According to the soil DNA extraction kit (PowerSoil^®^ DNA Isolation Kit) instruction manual steps for extracting DNA from rhizosphere soil, with a sample DNA concentration higher than 10 ng/μL. OD260/OD280 is between 1.8 and 2.0 to ensure DNA quality. The ITS general-purpose primers gITS7 (5 ‘GTGARTCATCGARTCTTTTG 3’) (Ihrmark et al., 2012) and ITS4 (5 ‘TCCTCCGCTTTATTGTGC 3’) (White et al., 1990) were selected for DNA amplification. PCR amplification system (50 μL) comprised the following: 10× PCR buffer, 5 μL; dNTPs, 4 μL; forward and reverse primers, 1 μL; Taq DNA polymerase, 0.5 μL; DNA sample, 1 μL; and ddH_2_O, 37.5 μL. Following PCR conditions were employed: pre-denaturation for 5 min at 94°C, denaturation for 30 s at 95°C, annealing at 56–62°C for 30 s, extension at 72°C for 30 s, fungal cycling for more than 35 steps, and finally extension at 72°C for 10 min. After separation and purification by 1% agarose gel electrophoresis, DNA concentration was detected using a NanoDrop 2000 (Thermo Fisher Scientific), and 30 samples were mixed according to the DNA concentration, such that the mixed samples contained 150 ng DNA. Finally, sequencing was performed using an Illumina MiSeq platform (Magigene, Guangdong).

### 2.3 Physicochemical properties of rhizosphere soil

After natural air drying, the soil samples were sieved, and the soil pH was measured using a pH meter (MP523-01) with a water-to-soil ratio of 2.5:1. Soil organic matter content was determined by potassium dichromate oxidation - external heating method; Kjeldahl method was used to determine soil total nitrogen (TN) content. The content of soil available phosphorus (AP) was determined by 0.5 M NaHCO_3_ extraction with molybdenum antimony resistance spectrophotometry. The determination of soil AK content by ammonium acetate extraction - flame photometric method (Nie and Wang, 2019).

### 2.4 Growth indexes of *P. ludlowii*

Leaf area, plant height, crown width, number of flowers, number of fruits, and fruit-setting rate were selected to characterize the growth of wild and cultivated *P. ludlowii* species. To determine the leaf area, five parietal lobules located 3–4 fully expanded to apical lobules upward from the base of flower branches were selected from each plant, and their maximum transverse and longitudinal diameters were measured. Additionally, five flowering branches were selected from each plant to determine the number of flowers and fruits per plant. The fruit-setting rate refers to the percentage of the number of fruits divided by the total number of flowers in *P. ludlowii* (number of fruits/number of flowers × 100%).

### 2.5 Statistical analysis

The sequencing results were uploaded to the Galaxy online analysis platform (http://mem.rcees.ac.cn:8080) (Feng et al., 2017), the FLASH tool was used to merge the same sequence upstream and downstream primers (Magoc and Salzberg, 2011), and Btrim was used to filter reads, to remove any sequences with degenerate bases and lengths less than 200 bp, retaining only 240–350 bp reads (Kong, 2011). Finally, UPARSE was used to remove chimeras and cluster sequences into operational taxonomic units (OTUs) in the sequences, with a 97% similarity threshold (Edgar, 2013). The total number of samples was 30; however, in this experiment, the number of reads obtained by sequencing one sample from LCT3 was < 10,000. After the second experiment, the sample was still disqualified; therefore, it was excluded from subsequent analysis. Based on the resulting 36,985 sequences, the OTU table was re-extracted, and species annotation was performed using the Unite database. Abundance and diversity indexes (Shannon, Simpson, and Chao1) were calculated, and based on Bray-Curtis distance, principal co-ordinate analysis (PCoA) and non-metric multidimensional scaling (NMDS) were used to analyze β diversity; Multi-Response Permutation Procedure (MRPP), analysis of similarities (ANOSIM), and permutational multivariate analysis of variance (PERMANOVA) were used to identify dissimilarities between groups. Mantel analysis and canonical correspondence analysis (CCA) were used to measure the relationship between the community structure and environmental factors. Fungi Functional Guild (FUNGuild) was used to predict the function of the rhizosphere soil fungal communities. All the analyses were performed using the Galaxy online analysis platform.

Using the Galaxy online platform (http://mem.rcees.ac.cn:8081), a molecular ecological network of the rhizosphere soil fungal communities of wild and cultivated species was constructed using the random matrix theory (RMT) method (Deng et al., 2012; Xiao et al., 2022). Topological roles, including within-module connectivity (*Zi*) and among-module connectivity (*Pi*), are classified as module hubs, network hubs, peripherals, or connectors (Olesen et al., 2007). The module hubs, network hubs, and many connectors in their respective modules are highly connected and can be considered as keystone species in the entire network (Deng et al., 2012). The results were visualized using Cytoscape 3.9.1 software (http://www.cytoscape.org/), and Student’s t-test was used to measure the significance between the empirical networks and random networks. Community assembly mechanisms were inferred using phylogenetic bin-based null model analysis on the Galaxy-iCAMP online platform (http://ieg3.rccc.ou.edu:8080) (Ning et al., 2020; Gu et al., 2022).

## 3 Results

### 3.1 Growth indexes of wild and cultivated *P. ludlowii* species

In terms of growth indicators for *P. ludlowii* (**Table 1**), the leaf area and crown width of the wild species showed significant increases with seasonal changes, whereas the plant height increased relatively gently, indicating that *P. ludlowii* exhibited vigorous growth in the original region. However, the leaf area of cultivated species only increased slowly with the change in season, and was significantly higher at LCT3 than at LCT1; therefore, the growth rate of *P. ludlowii* in the introduction region was relatively slow. Although the number of flowers and fruits of the wild species was significantly higher than that of the cultivated species, there was no significant difference in the fruit-setting rate between the two regions.

**Table 1:** Growth indexes of wild and cultivated *P. ludlowii* species.

Experimental field	Leaf area/cm^2^	Plant height/m	Crown width/m^2^	Number of flowers	Number of fruits	Fruit setting ratio/%
LCT1	426.55 ± 73.76Ab	2.31 ± 0.17Aa	2.14 ± 0.25Aa	21.99 ± 5.67B	/	/
LCT2	737.60 ± 62.32Aab	2.46 ± 0.14Aa	2.48 ± 0.20Ba	/	19.40 ± 4.04B	89.30 ± 7.36A
LCT3	851.00 ± 63.65Ba	2.49 ± 0.14Aa	2.53 ± 0.19Ba	/	/	/
LZT1	173.33 ± 114.42Bc	2.24 ± 0.25Ab	2.06 ± 0.33Ac	50.98 ± 17.62A	/	/
LZT2	586.49 ± 84.74Ab	2.44 ± 0.16Aab	2.67 ± 0.41Ab	/	47.20 ± 19.14A	91.18 ± 6.09A
LZT3	1216.01 ± 324.26Aa	2.61 ± 0.26Aa	3.21 ± 0.45Aa	/	/	/

Capital letters indicate different field in the same season, while lowercase letters indicate different seasons in the same field. The same below. '/' represents a null value.

### 3.2 Physicochemical properties of rhizosphere soil of wild and cultivated *P. ludlowii* species

The physicochemical properties of the rhizosphere soils of wild and cultivated peonies are shown in **Table 2**. The pH of the rhizosphere soil in the two regions was slightly acidic to nearly neutral, and the pH of the cultivated species was significantly greater than that of the wild species only at the flowering stage. The soil organic matter content was similar between the two regions; the organic matter content in both regions was the lowest at the flowering stage, and significantly increased to the maximum during the fruiting stage. The difference between the two regions was that during the autumn foliage stage, the content of soil organic matter of cultivated *P. ludlowii* species was reduced to a level similar to that at the flowering stage, whereas the soil organic matter content of wild species remained relatively unchanged from the fruiting stage to the autumn foliage stage. The AP content of the wild species was significantly higher than that of the cultivated species at different growth stages (*P<0.01*). The soil AP content of wild species gradually increased significantly with the growth period, whereas the AP content of cultivated species increased significantly from the flowering to fruiting stage and remained stable from the fruiting stage to autumn foliage stage. The variation trend of soil AK content in wild and cultivated species was consistent, both of which first increased and then decreased with the change in the growth period. Although the soil AK content of the wild species was higher than that of the cultivated species during all three growth periods, the difference was only significant during the autumn foliage stage.

**Table 2:** Physicochemical properties of rhizosphere soil of wild and cultivated *P. ludlowii* species.

Experimental field	pH	Organic matter/(g/kg)	TN/(g/kg)	AP/(mg/kg)	AK/(mg/kg)
LCT1	6.74 ± 0.41Aa	18.72 ± 3.28Ab	0.90 ± 0.21Aa	1.13 ± 0.05Bb	21.90 ± 3.46Ab
LCT2	6.59 ± 0.50Aa	24.08 ± 2.51Aa	1.03 ± 0.13Aa	1.40 ± 0.08Ba	32.68 ± 5.37Aa
LCT3	6.63 ± 0.25Aa	18.08 ± 4.87Bb	1.07 ± 0.30Aa	1.45 ± 0.13Ba	15.83 ± 2.49Bc
LZT1	5.91 ± 0.30Ba	18.57 ± 4.83Ab	1.05 ± 0.16Aa	1.23 ± 0.01Ac	27.78 ± 5.37Ab
LZT2	6.15 ± 0.16Aa	24.24 ± 2.27Aa	1.16 ± 0.19Aa	1.86 ± 0.05Ab	36.24 ± 3.54Aa
LZT3	6.19 ± 0.38Aa	24.81 ± 5.55Aa	1.13 ± 0.28Aa	2.02 ± 0.10Aa	23.74 ± 5.65Ab

TN, Total nitrogen; AP, Available phosphorus; AK, Available potassium. Capital letters indicate different field in the same season, while lowercase letters indicate different seasons in the same field.

### 3.3 Diversity and community structure of rhizosphere fungi in wild and cultivated *P. ludlowii* species

#### 3.3.1 Fungal diversity in rhizosphere soil

**Figure 1:**
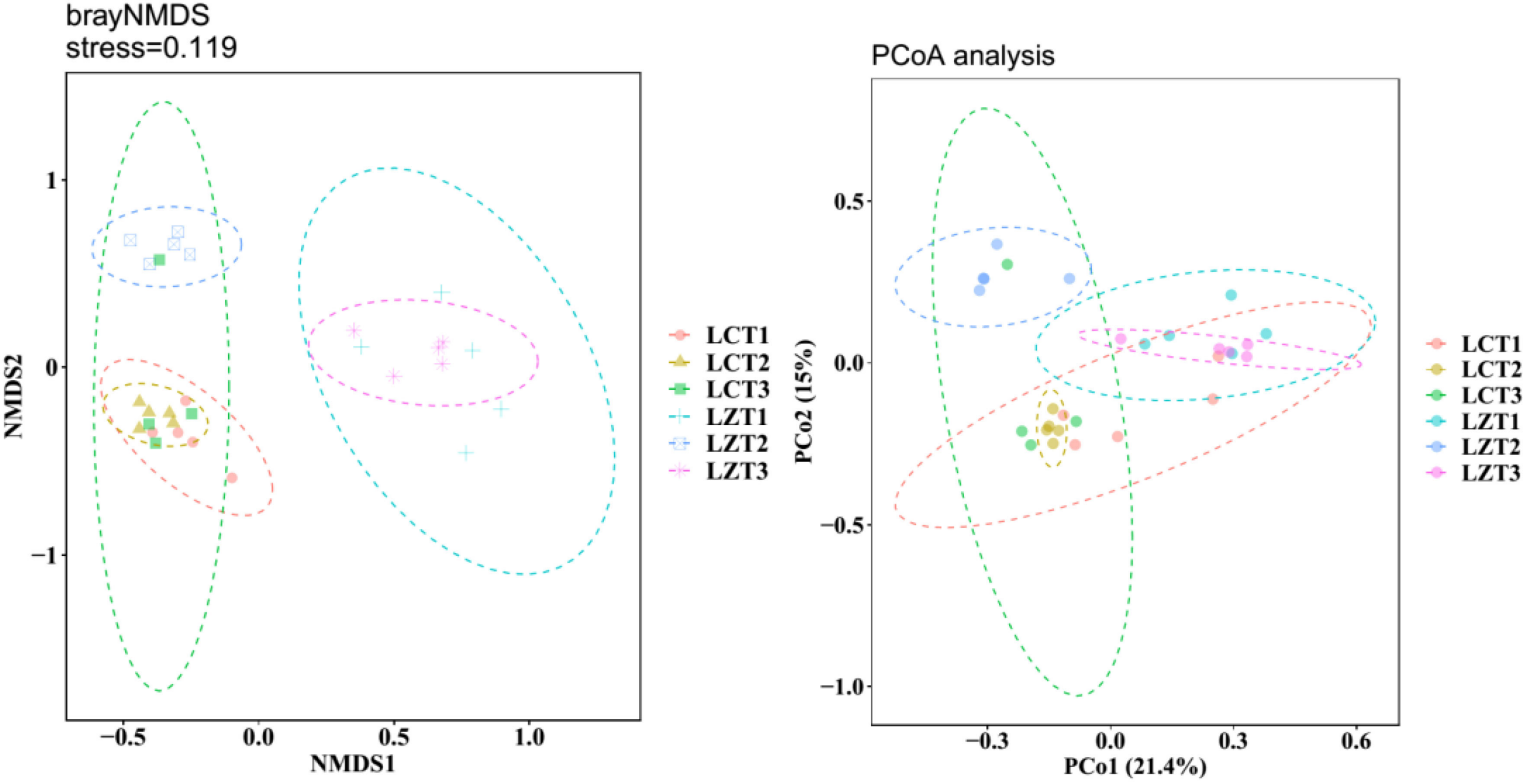
NMDS and PCoA analysis of the rhizosphere fungi community structure of *P. ludlowii*.

After re-extraction, all samples were clustered into 4,162 OTUs. The rarefaction curves (**Figure S3**) showed that as the sequence number increased, the rarefaction curves of each sample tended to flatten, indicating that the sampling was reasonable and accurately reflected the fungal community in the rhizosphere soil of *P. ludlowii*. During the same growth period, the Simpson’s evenness and Chao1 indexes of the two regions did not show significant changes, and the Shannon index was significantly higher than that of the cultivated species only at the fruiting stage (**Table S1**). The results of NMDS analysis based on the Bray–Curtis distance matrix revealed (**Figure 1**) that the rhizosphere soil fungal community structure of wild and cultivated species was similar during the flowering stage, but significantly different during the fruiting and autumn foliage stages (*P<0.05*). The fungal community structure remained similar among the three cultivation periods, whereas in the wild species, the community structure of LZT2 was significantly different from that of LZT1 and LZT3. Simultaneously, PCoA (**Figure 1**) and dissimilarity tests (**Table S2**) were performed based on the Bray–Curtis distance matrix of the rhizosphere soil fungal community, which were consistent with the NMDS results.

#### 3.3.2 Taxonomy of rhizosphere soil fungi

**Figure 2:**
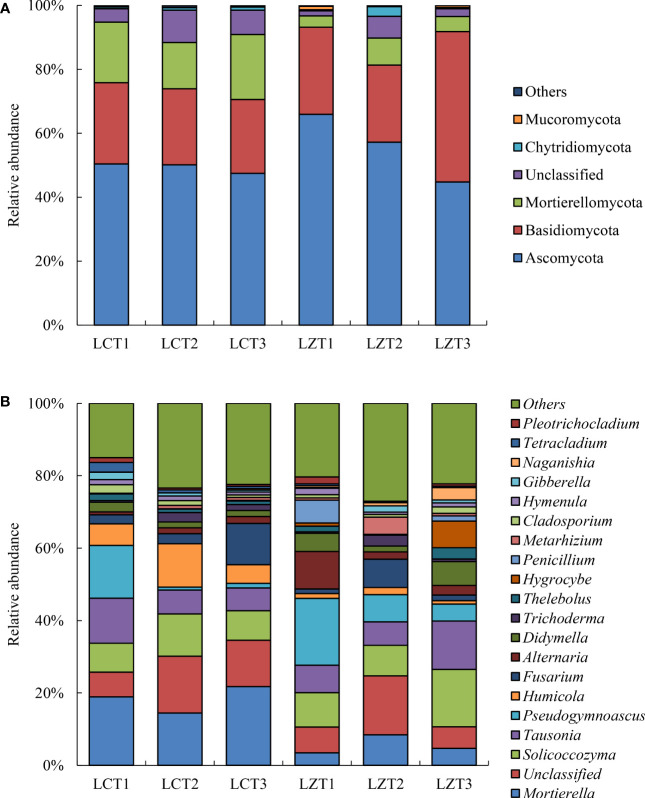
Relative abundance of rhizosphere fungi community at the phylum level **(A)** and genus level **(B)** of *P. ludlowii*.

After annotation using the UNITE database, all samples were divided into 18 phyla and 719 genera. The dominant fungi with a relative abundance >1% at the phylum level are shown in **Figure 2A**. Because of the uneven classification at the genus level, many fungi with a relative abundance >1% were present, and only the top 20 dominant fungi genus were selected for analysis (**Figure 2B**).

Among the rhizosphere soil fungal communities of the wild species, Ascomycota was the most abundant phylum at the flowering stage, but its relative abundance gradually decreased as the plants grew, reaching its lowest abundance during the autumn foliage stage of *P. ludlowii*; whereas, Basidiomycota significantly increased during the autumn foliage stage and became the most dominant phylum of the wild *P. ludlowii* species. At the fruiting stage, the relative content of Chytridiomycota reached its maximum abundance (2.99%), which was significantly higher than that at the flowering (0.36%) and autumn foliage stages (0.33%). In the cultivated species, Ascomycota did not change with the growth period and remained the most dominant fungi with the highest relative content, whereas other phyla remained relatively stable in their content. During the flowering stage of *P. ludlowii*, the relative abundance of Ascomycota in wild species was significantly higher than that in the cultivated species, whereas the relative abundance of Mortierellamycota and Mucoromycota were significantly lower than those in the cultivated species; conversely, the relative abundance of Chytridiomycota in wild species was significantly higher than that in the cultivated species at the fruiting stage.

The relative abundances of rhizosphere soil fungi at the genus level differed between the wild and cultivated species, and the variations were complex. The relative abundance of *Mortierella* in the cultivated species (14.44–21.74%) was significantly higher than that in the wild species (3.47%–8.47%) at all three growth stages. The relative abundances of *Solicoccozyma* and *Didymella* first decreased and then increased with the growth period of the wild species, reaching higher levels (15.91% and 6.55%) during the autumn foliage stage, which were also significantly higher than those of cultivated species during the same period. The relative abundance of *Pseudogymnoascus* was the highest during the flowering stage but decreased afterwards, exhibiting a similar trend of change in both regions. The relative abundance of *Humicola* was significantly higher in the LCT2 stage than that in the other stages and was also significantly higher than that in the LZT2 stage. Similarly, the relative abundance of *Thelebolus* in LZT3 was significantly higher than that in LZT1 and LZT2, and significantly higher than that in LCT3 at the same time. *Hymenula* was present in a stable manner in cultivated species; however, in wild species, it decreased initially and then increased with the growth period, and its relative abundance at the fruiting stage was significantly lower than that during the other stages. The relative abundance of *Tetracladium* in LCT1 was significantly higher than that in LCT2 and LCT3, also significantly higher than that in LZT1 at the same time.

### 3.4 Functional prediction of rhizosphere fungi communities and analysis of environmental factors in wild and cultivated *P. ludlowii* species

#### 3.4.1 Functional prediction

FUNGuild was used to predict the trophic modes and functional groups of the rhizosphere soil fungal communities of the wild and cultivated species of *P. ludlowii*, resulting in the classification of seven trophic modes (**Table 3**). These were mainly Pathotrophs, Pathotroph-Saprotrophs, Saprotrophs, and Saprotroph-Symbiotrophs, accounting for 9.96–25.44%, 7.93–16.53%, 33.51–47.25%, and 16.73–40.80%, respectively. In mixed Pathotroph-Saprotrophs, LZT1 and LZT3 were significantly higher than LCT1 and LCT3, respectively. Further classification of Pathotrophs, Saprotrophs and Symbiotrophs according to guild types (**Figure S4**) revealed minimal differences between wild and cultivated species. However, the abundance of Soil Saprotroph in LCT2 was significantly higher than that in LZT2, and Mixed Saprotroph in LCT1 was significantly higher than that in LZT1.

**Table 3:** Prediction of trophic mode function of rhizosphere fungi of wild and cultivated *P. ludlowii* species.

Trophic mode	LCT1	LCT2	LCT3	LZT1	LZT2	LZT3
Pathotrophs	9.96% ± 0.05Aa	14.25% ± 0.06Aa	10.17% ± 0.05Aa	12.96% ± 0.07Aa	25.44% ± 0.23Aa	7.26% ± 0.02Aa
Pathotroph-Saprotrophs	8.23% ± 0.06Ba	8.62% ± 0.01Aa	9.24% ± 0.02Ba	16.50% ± 0.08Aa	7.93% ± 0.02Ab	16.53% ± 0.04Aa
Pathotroph-Saprotroph-Symbiotrophs	0.51% ± 0.00Ab	5.32% ± 0.05Aa	3.15% ± 0.02Aab	0.47% ± 0.00Ab	5.11% ± 0.05Aa	0.63% ± 0.01Ab
Pathotroph-Symbiotrophs	1.10% ± 0.02Aa	1.78% ± 0.02Aa	0.51% ± 0.00Aa	0.35% ± 0.00Aa	0.90% ± 0.00Aa	1.08% ± 0.01Aa
Saprotrophs	43.17% ± 0.21Aa	33.80% ± 0.12Aa	33.51% ± 0.14Aa	47.25% ± 0.20Aa	39.48% ± 0.19Aa	42.37% ± 0.15Aa
Saprotroph-Symbiotrophs	36.29% ± 0.24Aa	32.46% ± 0.11Aa	40.80% ± 0.21Aa	16.73% ± 0.06Aa	18.82% ± 0.14Aa	27.03% ± 0.17Aa
Symbiotrophs	0.74% ± 0.01Aa	3.77% ± 0.04Aa	2.61% ± 0.02Aa	5.74% ± 0.04Aa	2.32% ± 0.02Aa	5.10% ± 0.08Aa

Capital letters indicate different field in the same season, while lowercase letters indicate different seasons in the same field.

#### 3.4.2 Mantel analysis and CCA

**Figure 3:**
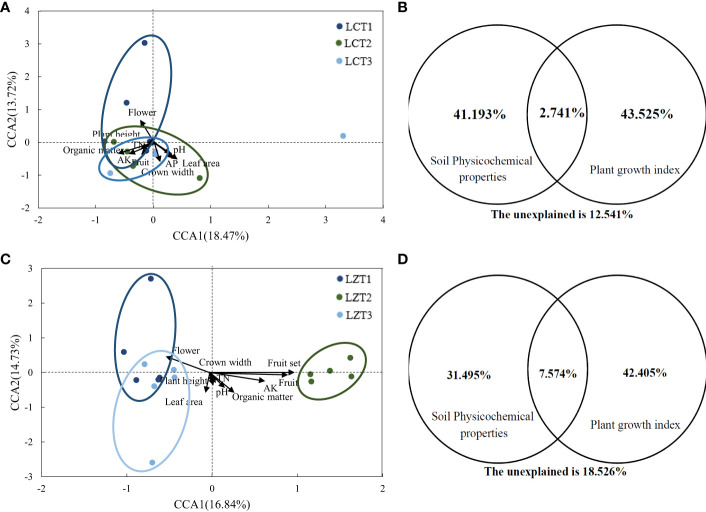
CCA and CCA-VPA analysis of the rhizosphere fungi community of *P. ludlowii* **(A, B)**: LC; **(C, D)**: LZ.

Mantel correlation analysis was conducted based on the Bray–Curtis distance matrix of the rhizosphere soil fungal communities, along with the growth indexes and soil physicochemical properties of *P. ludlowii* (**Table S3**). The rhizosphere soil fungal community structure of cultivated species showed a significant positive correlation only with the soil organic matter content (*P<0.01*), whereas the rhizosphere soil fungal community structure of wild species was significantly correlated with the number of flowers, number of fruits, and fruit-setting rate as growth indicators of *P. ludlowii*. Further evaluation of the potential relationship between the rhizosphere soil fungal communities of the wild and cultivated species and growth and soil physicochemical properties of *P. ludlowii* was performed using CCA (**Figures 3A, C**), with explanatory ratios of 28.59% and 31.57%, respectively. The first axis of the cultivated species showed a positive correlation with pH, AP, leaf area, and crown width and a negative correlation with organic matter, TN, AK, plant height, number of flowers, and number of fruits, with organic matter(*P=0.011*) and leaf area (*P=0.017*) showing stronger correlations. The first axis of the wild species was positively correlation with pH, organic matter, TN, AK, plant height, and crown width and negatively correlation with leaf area and number of flowers, with AK(*P=0.026*) and number of flowers (*P=0.020*) showing stronger correlations. According to CCA-VPA (**Figures 3B, D**), in cultivated species, the total interpretation rate of all factors affecting plant growth indexes and soil physicochemical properties was 87.46%, with the two factors contributing close to 42% of the interpretation rate, and a co-interpretation rate of 2.74%. In the wild species, the interpretation rate of plant growth indexes was higher than that of soil physicochemical properties, and the co-interpretation rate of 7.57% was also higher than that of cultivated species.

### 3.5 Community assembly mechanisms and molecular ecological networks of rhizosphere soil fungi of wild and cultivated *P. ludlowii* species

#### 3.5.1 Community assembly mechanisms

**Figure 4:**
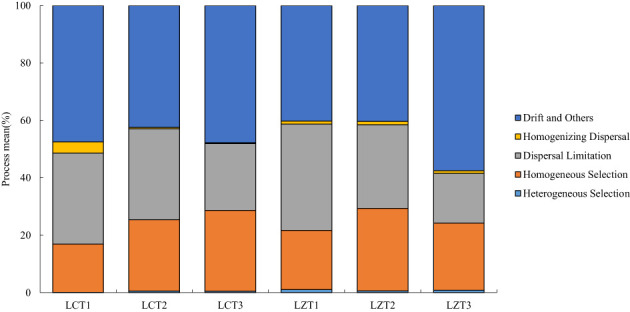
Ecological processes of the rhizosphere fungal community assembly of *P. ludlowii*.

To gain a better understanding of the ecological processes driving the assembly of rhizosphere soil fungal communities in the wild and cultivated species, iCAMP was used to infer these processes from phylogenetic data (**Figure 4**). The results revealed that the most important ecological process driving rhizosphere soil fungal community assembly of both wild and cultivated species was drift, and there was no significant difference between the two *P. ludlowii* species. However, in the wild species, the LZT3 stage exhibited a significantly higher importance than did the other stages. Furthermore, the ecological processes of both wild and cultivated species are dispersal limitation and homogeneous selection. In the LZ, only the dispersal limitation of LZT1 gradually decreased, and was significantly higher than that of LZT3.

#### 3.5.2 Molecular ecological networks

**Figure 5:**
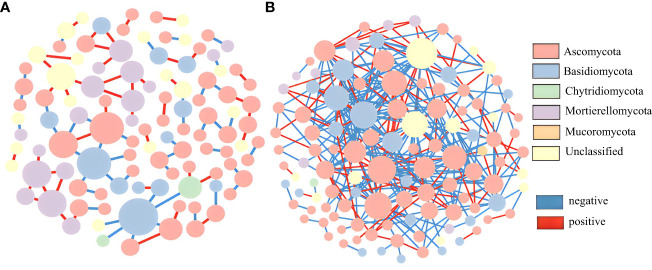
Molecular ecological network of the rhizosphere fungi of *P. ludlowii* **(A)**: LC; **(B)**: LZ) A node represents an OTU; The color of nodes represents gate horizontal classification, and the size of nodes represents the number of connections; Each line represents a correlation between two nodes connected to it, with red representing a positive correlation and blue representing a negative correlation.

In this study, OTU tables of the wild and cultivated species from the three stages were combined to analyze their fungal molecular networks. Co-occurring OTUs in 50% of the samples were screened and retained, and the same cutoff value (0.84) was selected. The wild species had significantly more total nodes with higher avgK and avgCC values than those of the cultivated species (**Table S3**). These network topological parameters indicate that wild species have more complex molecular network modules than do cultivated species. For both the wild and cultivated species, most of the fungal network nodes were peripherals and there were no network hubs (**Figure 5**). The cultivated species had only one module hub belonging to Basidiomycota. Although the wild species had no module hubs, there were 17 connectors: 15 belonging to Ascomycota and one each unclassified and belonging to Basidiomycota (**Figure S5**). Therefore, Ascomycota may be a key fungal group in the rhizosphere soil of the wild *P. ludlowii* species, but there were no key fungal groups in the cultivated species.

## 4 Discussion

### 4.1 Growth index and soil physicochemical properties of wild and cultivated *P. ludlowii* species

In this study, the leaf area of LCT1 at the flowering stage was greater than that of LZT1 at the same stage, whereas the leaf area of the cultivated species increased slowly during the fruiting and autumn foliage stages. However, the wild species had a smaller leaf area before flowering, but showed the fastest growth rate from the flowering stage to fruiting stage, and still maintained a higher growth rate after the fruiting stage. Plants require more nutrients during the flowering stage and their roots absorb them vigorously, resulting in the lowest soil organic matter content and similar rhizosphere soil fungal community structures in the two regions.

The study revealed that the soil organic matter content of cultivated species varied significantly and was lowest at the flowering stage, increased significantly at the fruiting stage, and decreased to a similar level during the autumn foliage stage. The Mantel analysis revealed that only soil organic matter of the cultivated species significantly and positively correlated with the rhizosphere soil fungal community structure. In a study of rhizosphere soil microorganisms at the introduction region by Gao et al. (2022), soil pH was significantly positively correlated with rhizosphere soil bacteria, and soil organic matter, ammonium nitrogen, and AK content were significantly positively correlated with the AMF community but had no significant influence on the rhizosphere soil fungal communities. The difference in results may be due to differences in sampling time and soil layers. In this study, the sampling time was divided into three periods with a mixed soil layer of 0–20 cm, whereas the research conducted by Gao et al. (2022) included only stratified sampling of 0–10 cm and 10–20 cm in August. By decomposing complex plant residues, Mortierellomycota and Mucoromycota can increase soil phosphorus content and promote plant growth (Zhang et al., 2021; Fan et al., 2022). At the flowering stage of *P. ludlowii*, soil AP content of cultivated species was significantly lower than that of wild species, whereas the relative abundances of Mortierellomycota and Mucoromycota were significantly higher than those of the wild species. Such a contradictory conclusion may be explained by the following: (1) Due to the low phosphorus content in the mountainous soil at the introduction region, to meet the demand of phosphorus for plant growth, *P. ludlowii* may have recruited more Mortierellomycota and Mucoromycota to break down plant residues and release phosphorus. However, given the lack of soil background data before introduction, further research is required. (2) According to the research group’s investigation, there were few species of weeds in the introduced region, mainly *Erigeron annuus* (L.) Pers., *Artemisia* L. At the same time, weeds were removed every summer, and there were 78 species of accompanying plants of original habitat (Ni, 2009). Therefore, we concluded that the phosphorus content produced by the decomposition of Mortierellomycota and Mucoromycota was low, leading to a low AP content at the introduced region. Additionally, it was found that the soil AP of cultivated species was positively correlated with the rhizosphere soil fungal community structure, Therefore, low AP content at the introduced region is speculated to have affected plant growth, and the two types of fungi, Mortierellomycota and Mucoromycota, play an important role in promoting phosphorus absorption by the cultivated *P. ludlowii* species.

### 4.2 Rhizosphere soil fungi community of wild and cultivated *P. ludlowii* species

The effects of wild and cultivated species on the diversity of rhizosphere soil microbial communities are inconsistent. Compared to wild *Agave salmiana*, cultivated *Agave tequilana* has lower microbial diversity (Coleman-Derr et al., 2016), whereas other studies have shown that introduction and cultivation can improve the diversity of fungal communities in rice (Chang et al, 2021). In this study, although the α diversity of the cultivated species did not decrease compared with that of the wild species, the β diversity of the cultivated species was significantly different. Ascomycota and Chytridiomycota are mainly saprotrophic that, decompose organic matter in the soil into ionic states that can be absorbed by plants to help improve soil fertility (James et al., 2006; Beimforde et al., 2014). Among the cultivated species, Ascomycota consistently had the highest relative abundance and played an important role in decomposing dead leaves in introduced regions, providing nutrients for the growth of *P. ludlowii* (Gao et al., 2022).

In the rhizosphere soil fungal community of the wild species *P. ludlowii*, Ascomycota had the highest relative abundance only at the flowering stage; however, according to a molecular network analysis (Jiang et al., 2017), Ascomycota is the key group of the rhizosphere soil fungal community. Chytridiomycota had the highest relative abundance (2.99%) at the fruiting stage, which was significantly higher than that of the cultivated species. As a perennial flowering plant, *P. ludlowii* requires more nutrients during its flowering and fruiting stages. These two fungi decomposed dead branches, leaves, and other complex organic substances for plant absorption and utilization at different growth stages. Basidiomycota significantly increased in the autumn foliage stage, and became the most dominant species of the wild species *P. ludlowii* (Lu et al., 2020a), mainly due to the significant increase in *Solicoccozyma*, which contributed to improving the accumulation of medicinal components in the roots of the wild species *P. ludlowii*; it is speculated that the medicinal properties of the cultivated species *P. ludlowii* would be weakened (Schmidt-Dannert, 2016). The relative abundance of *Mortierella* in the cultivated species was significantly higher than that of the wild species, and the relative abundance of *Humicola* in LCT2 was significantly higher than that during the other stages and was also significantly higher than that in LZT2. Some *Mortierella* and *Humicola* fungi have biological control functions that can inhibit pathogenic bacteria such as *Fusarium oxysporum*, *Alternaria brassicicola*, and *Phytophthora capsici* (Ko et al., 2011; Edgington et al., 2014; Li et al., 2014; Yang et al., 2014). Consequently, the cultivated *P. ludlowii* species may be more susceptible to some pathogens, especially during the fruiting stage, *P. ludlowii* enhances its ability to resist pathogens by recruiting *Mortierella* and *Humicola* fungi. The relative abundance of *Pseudogymnoascus* was the highest during the flowering stage and then decreased significantly. This genus can decompose cellulose, which is beneficial for degrading dead branches and leaves, and improving the content of soil organic matter (Thormann, 2011). *Pseudogymnoascus* fungi are enriched during the flowering stage of *P. ludlowii* because of the high nutrient demand at this stage, thus becoming the most dominant genus in both the wild and cultivated species.

In mixed Pathotroph-Saprotrophs, LZT1 and LZT3 were significantly higher than LCT1 and LCT3. Mixotrophic fungi exhibit a diverse fungal functional profile, that is conducive to maintaining ecological stability (Fan et al., 2022). Thus, wild species have more stable fungal ecosystems than cultivated species. Although there was little difference between Pathotrophs in the wild and cultivated species, the Soil Saprotroph of LCT2 was significantly higher than that of LZT2, and the Mixed Saprotroph of LCT1 was significantly higher than that of LZT1. These Saprotrophs are prone to enrichment in decaying and diseased plants and are more prone to causing plant diseases (Zhang et al., 2022). Rhizosphere soil fungi and bacteria exhibited similar patterns, compared to cultivated varieties, wild beet (*Beta vulgaris* ssp.) and wild soybean (*Glycine soja* Sieb. et *Zucc*) have diverse rhizosphere microbial communities, and microbial communities isolated from native soil have antibacterial activities that can protect plants against environmental stress (Zachow et al., 2014; Ma et al., 2019).

### 4.3 Compared to cultivated species, rhizosphere soil fungi of wild species exhibit more intricate molecular ecological networks

The aggregation structure of rhizosphere soil fungal communities was similar between wild and cultivated species and was dominated mainly by stochastic processes. The most important ecological process was drift (40.28–57.55%), followed by dispersal limitation (17.28–37.00%), with no significant differences. Drift is a stochastic process in the soil microbial community caused by factors such as reproduction and gradual death (Zhou and Ning, 2017). Dispersal limitation increases community variation and diversifies the microbial community structure (Stegen et al., 2013). Both are important ecological assemblages that drive soil fungi in multiple regions (Aslani et al., 2021; Gu et al., 2022). The difference is that drift and dispersal limitations exist stably in the cultivated species, whereas the opposite trend is observed in wild species. Drifts gradually increased with the growth period, whereas dispersal limitation gradually decreased, showing a significant difference from LZT1 to LZT3. However, the specific reasons for this phenomenon require further exploration. Homogeneous selection (16.84–28.79%) drove the deterministic ecological process of the fungal communities of wild and cultivated species, which enabled fungal communities to maintain a stable state after being disturbed by the environment, and the community structure was more similar (Dini-Andreote et al, 2015).

In this study, ecological network analysis was used to visualize the interactions between rhizosphere soil fungi in wild and cultivated species (Fuhrman, 2009). Based on the module network topology parameters, wild species exhibited more complex molecular network modules than did the cultivated species, indicating that wild species of *P. ludlowii* can respond more stably to changes in the external environment. Although the growth of the cultivated *P. ludlowii* species did not show a significant decline in this study, in the long run, as harmful fungi increase, beneficial fungi decrease, and network connections between fungi decline, plant–soil negative feedback is likely to occur (Martín-Robles et al., 2020). Studies have reported that the introduction and domestication of many food crops such as soybean, rice, and tomato, reduce network complexity and plant resistance to pathogenic fungi, resulting in microbe-mediated soil negative feedback (Carrillo et al., 2019; Chang et al., 2020; Chang et al., 2021).

## 5 Conclusion

Both wild and cultivated species of *P. ludlowii* can bloom and bear fruit normally, and the physicochemical properties of the soil are similar. However, the number of flowers, number of fruits, and soil AP and AK contents of the wild species were higher than those of the cultivated species. The diversity of the rhizosphere soil fungi was similar between the wild and cultivated species, but their community structures differed during the fruiting and autumn foliage stages. The structure of the rhizosphere soil fungal community of the cultivated species was influenced by *P. ludlowii* growth and soil physicochemical properties, whereas the growth of the wild species was more closely affected by the growth of *P. ludlowii*. The dominant fungal phyla in both the wild and cultivated species were Ascomycota and Basidiomycota, which play important roles in the growth of *P. ludlowii*. There was a significant difference in the dominant fungal genera between the wild and cultivated species, whereas in the cultivated species, it was relatively small, with the relative abundance of *Mortierella* being significantly higher in the cultivated species than that in the wild species. *Humicola* abundance was significantly higher in the LCT2 stage than during the other stages and was also significantly higher than that in the LZT2 stage. In the wild species, the relative abundances of *Solicoccozyma*, *Didymella* and *Thelebolus* reached higher levels during the autumn foliage stage. The aggregation structure of the fungal community in the rhizosphere soil of the wild and cultivated species was controlled by stochastic processes, and there were no significant differences. However, the abundance of pathogenic fungi in the cultivated species was relatively high and the network connection among fungi was weakened, making it more susceptible to plant diseases. The wild species not only had more mixotrophic fungi but also had more complex molecular network modules, which are more conducive to resisting external environmental interference and maintaining the relative stability of the system.

## Data availability statement

The datasets for this study can be found in the NCBI-SRA database, accession number PRJNA943533.

## Author contributions

HYQ: Laboratory experiment analysis, data analysis, paper writing. GDL: Field sampling and laboratory analysis, paper improvement. TY: Conception, paper refining and improvement. All authors contributed to the article and approved the submitted version.
