# Supplementary material for: Differences in rhizosphere soil fungal communities of wild and cultivated Paeonia ludlowii species
Source: Front Plant Sci. 2023 Sep 11;14:1194598. doi: 10.3389/fpls.2023.1194598 (PMC10520497; doi:10.3389/fpls.2023.1194598)
Supplement: Supplementary file 1 [file DataSheet_1.docx]

Supplementary Material

**Differences in rhizosphere soil fungal communities between wild and cultivated *Paeonia ludlowii* species**

Hongyong Qiao^1^, Danlei Gao^1,2^, Tao Yuan^1*^

*^1^ Beijing Key Laboratory of Ornamental Plants Germplasm Innovation & Molecular Breeding, National Engineering Research Center for Floriculture, Beijing Laboratory of Urban and Rural Ecological Environment, Key Laboratory of Genetics and Breeding in Forest Trees and Ornamental Plants of Ministry of Education, School of Landscape Architecture, Beijing Forestry University, Beijing China*

*^2^ Science and Technology Development Center, National Forestry and Grassland Administration, Beijing, China*

***Correspondence:** E-mail: yuantao@bjfu.edu.cn

**Detailed information of the Supplementary Material**

The number of Figures: 5

The number of Tables: 4

1. **Figures**

Figure S1 Original Habitat of *P. ludlowii*

Figure S2 Introducing region of *P. ludlowii*

Figure S3 Rhizosphere fungi rarefaction curves of *P. ludlowii*

Figure S4 Prediction of guild function of rhizosphere soil fungi in wild and cultivated *P. ludlowii* species

Figure S5 Fungi module nodes topology role distribution in rhizosphere soil of *P. ludlowii*


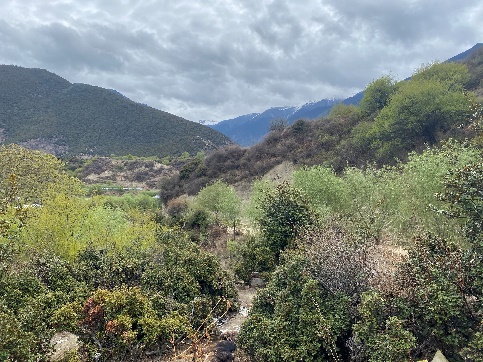

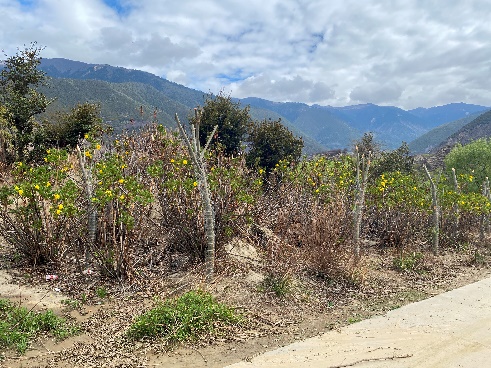


Figure S1


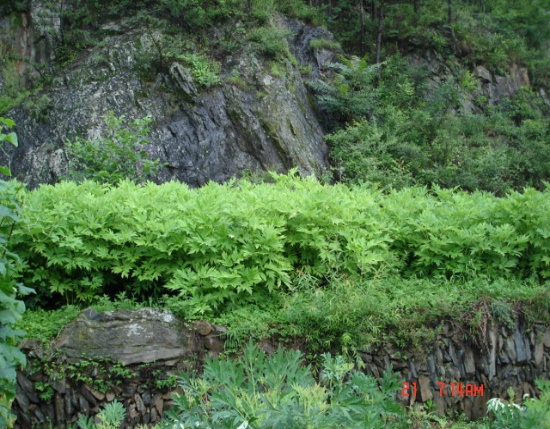

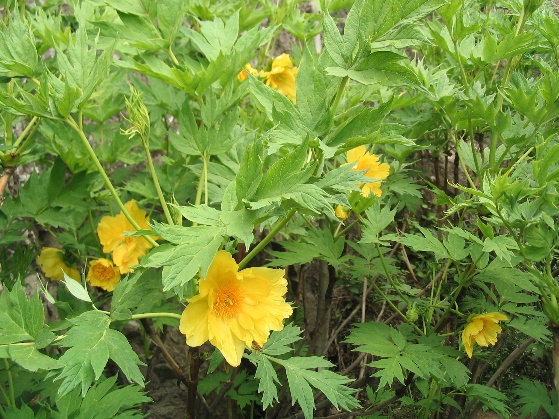


Figure S2

Figure S3

Figure S4


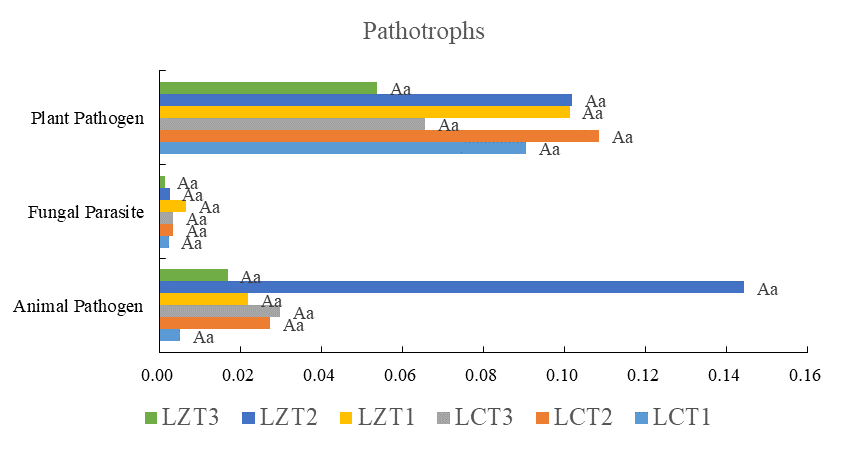


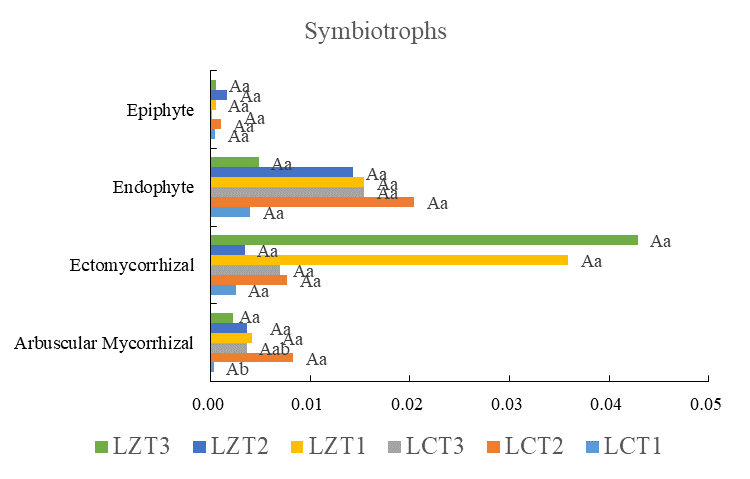


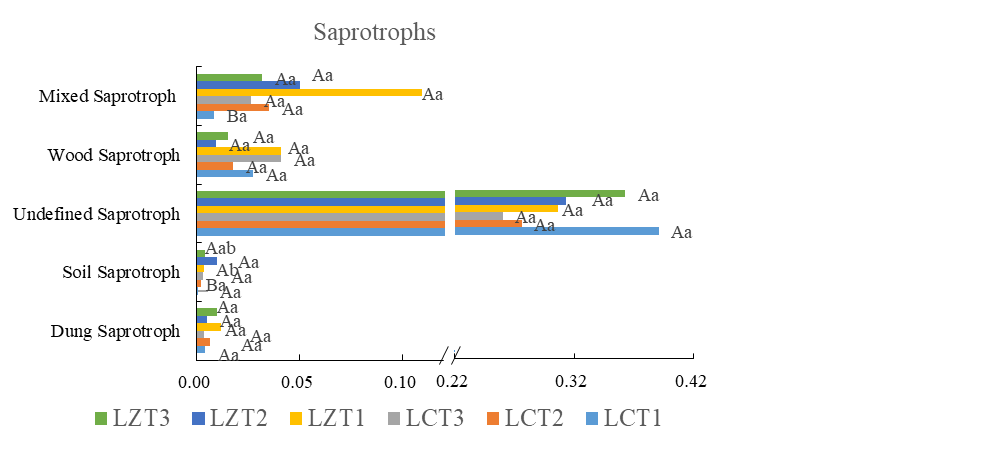


Figure S5


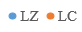


1. **Table**

| Table S1 α-diversity of rhizosphere fungi of *P. ludlowii* | | | |
| --- | --- | --- | --- |
| Experimental field | Shannon | Simpson evenness | chao1 |
| LCT1 | 3.42±0.82Aa | 0.04±0.02Aa | 506.95±257.85Aa |
| LCT2 | 4.03±0.47Ba | 0.05±0.04Aa | 532.64±90.60Aa |
| LCT3 | 4.08±0.36Aa | 0.05±0.03Aa | 561.92±139.02Aa |
| LZT1 | 3.22±0.85Ab | 0.03±0.03Aa | 455.86±148.72Aa |
| LZT2 | 4.17±0.64Aa | 0.05±0.03Aa | 613.01±181.03Aa |
| LZT3 | 3.59±0.16Aab | 0.03±0.01Aa | 569.01±123.31Aa |

| Table S2 The dissimilarity test of fungal community structure based on Bray–Curtis distance of *P. ludlowii* | | | | | | |
| --- | --- | --- | --- | --- | --- | --- |
| Dissimilarity result of Bray-Curtis distance | MRPP | | ANOSIM | | PERMANOVA | |
|  | Delta | *P* | R | *P* | F | *P* |
| LCT1 VS. LCT2 | 0.6072 | 0.146 | 0.168 | 0.117 | 1.4259 | 0.114 |
| LCT1 VS. LCT3 | 0.6257 | 0.193 | 0.1812 | 0.088 | 1.4184 | 0.137 |
| LCT1 VS. LZT1 | 0.6477 | 0.073 | 0.368 | 0.042* | 1.9756 | 0.061 |
| LCT2 VS. LCT3 | 0.6252 | 0.87 | -0.1 | 0.726 | 0.791 | 0.752 |
| LCT2 VS. LZT2 | 0.5823 | 0.012* | 0.948 | 0.008** | 3.9779 | 0.012* |
| LCT3 VS. LZT3 | 0.5804 | 0.021* | 0.6437 | 0.01** | 3.3986 | 0.016* |
| LZT1 VS. LZT2 | 0.6197 | 0.008** | 0.864 | 0.004** | 4.4842 | 0.01** |
| LZT1 VS. LZT3 | 0.6039 | 0.164 | 0.048 | 0.287 | 1.2882 | 0.021* |
| LZT2 VS. LZT3 | 0.542 | 0.013* | 0.956 | 0.011* | 5.9676 | 0.008** |

| Table S3 Mantel analysis of rhizosphere soil fungal community structure, plant growth index and soil physicochemical properties in wild and cultivated *P. ludlowii* species | | | | | |
| --- | --- | --- | --- | --- | --- |
| Type | Index | LC | | LZ | |
|  |  | r.BC | p.BC | r.BC | p.BC |
| Plant growth index | Leaf area/cm^2^ | 0.1869 | 0.102 | -0.0258 | 0.541 |
|  | Plant height/m | -0.3366 | 0.975 | -0.0723 | 0.712 |
|  | Crown width/m^2^ | 0.0848 | 0.287 | -0.114 | 0.875 |
|  | Number of flowers | 0.206 | 0.135 | 0.2144 | **0.049** |
|  | Number of fruits | -0.0948 | 0.686 | 0.5645 | **0.002** |
|  | Fruit set ratio/% | -0.0511 | 0.584 | 0.6936 | **0.001** |
| Soil physicochemical properties | pH | 0.1178 | 0.223 | 0.0704 | 0.281 |
|  | Organic matter | 0.5334 | **0.003** | -0.0435 | 0.627 |
|  | TN | 0.0854 | 0.294 | -0.1337 | 0.883 |
|  | AP | 0.1246 | 0.181 | 0.1007 | 0.17 |
|  | AK | 0.0879 | 0.251 | 0.1768 | 0.067 |

| Table S4 Comparison of topological characteristics between empirical network and random network of rhizosphere soil fungi in wild and cultivated *P. ludlowii* species | | | | | | | | | | | | |
| --- | --- | --- | --- | --- | --- | --- | --- | --- | --- | --- | --- | --- |
| Experimental field | Empirical network | | | | | | | | Random network | | | |
|  | Cutoff | Total nodes | Total links | R^2^ of power-law | Average degree  (avgK) | Average clustering coefficient  (avgCC) | Average path distance  (GD) | Modularity  (module number) | Average clustering coefficient  (avgCC) | Average path distance  (GD) | Modularity |  |
| LZ | 0.84 | 113 | 322 | 0.908 | 5.699 | 0.179 | 2.923 | 0.399(14) | 0.041±0.013** | 2.879±0.047** | 0.336±0.009** |  |
| LC | 0.84 | 104 | 86 | 0.949 | 1.654 | 0.087 | 3.339 | 0.907(26) | 0.001±0.004** | 5.189±0.048** | 0.860±0.014** |  |
| Note: ** represents a very significant difference between empirical network attributes and random network attributes of the same index (*P<0.001*) | | | | | | | | | | | | |
